# Alcohol intake aggravates adipose browning and muscle atrophy in cancer-associated cachexia

**DOI:** 10.18632/oncotarget.22243

**Published:** 2017-11-01

**Authors:** Bo Wang, Faya Zhang, Hui Zhang, Zhixiu Wang, Yan-Nan Ma, Mei-Jun Zhu, Min Du

**Affiliations:** ^1^ Beijing Advanced Innovation Center for Food Nutrition and Human Health, Beijing 100094, P. R. China; ^2^ Department of Animal Sciences, Washington State University, Pullman, WA 99164, USA; ^3^ Department of Pharmaceutical Sciences, College of Pharmacy, Washington State University, Spokane, WA 99210, USA; ^4^ School of Food Science, Washington State University, Pullman, WA 99164, USA; ^5^ Department of Chemistry and Lifer Sciences, Gansu Normal University for Nationalities, Hezuo 747000, P. R. China

**Keywords:** alcohol, cachexia, adipose browning, muscle atrophy, retinoic acid

## Abstract

Cancer is commonly associated with cachexia, a paraneoplastic syndrome characterized by body weight loss, muscle wasting, adipose tissue atrophy and inflammation. Chronic alcohol consumption increases the risk of multiple types of cancer, and enhances cancer-associated cachexia (CAC), but the underlying mechanisms remain poorly defined. To test, C57BL/6 mice were fed with 0% or 20% (w/v) alcohol for 3 months, then inoculated with B16BL6 melanoma cells subcutaneously in the right side of the hip and continued to feed with/without alcohol for 3 or 4 weeks. Alcohol intake upregulated ALDH1A1 expression and elevated retinoic acid (RA) content in inguinal white adipose tissue (iWAT), which led to enhanced iWAT browning and brown adipose tissue (BAT) activation, accelerating fat loss. Moreover, alcohol increased muscle loss through augmenting muscle protein degradation, cell apoptosis and inflammation. In addition, alcohol reduced satellite cell density and impaired myogenesis in skeletal muscle. Taken together, alcohol aggravates cancer-associated cachexia at least partially through elevating adipose browning and muscle atrophy.

## INTRODUCTION

Cancer-associated cachexia (CAC) is a paraneoplastic syndrome characterized by body weight loss, muscle wasting, adipose tissue atrophy and inflammation [1–3]. Cachectic patients progressively loss muscle and fat mass [2], which is associated with increased mortality risk [4]. Up to now, CAC is regarded as a non-curable condition [5] and there are very limited options to slow down CAC [6]. Because cachectic patients have reduced food intake [6], nutritional supplementary therapy partially reverses the wasting associated with cachexia [5], but the effectiveness is limited, suggesting the existence of additional mechanisms besides nutrition which are responsible for CAC incidence.

Increased metabolic rate, which accelerates weight loss, is one of major causes of CAC [7]. Due to the uncoupling effect of uncoupling protein-1 (UCP-1) which dissipates cross-membrane electrical gradient as heat, white adipose tissue browning increases energy expenditure [8]. Brown adipose tissue (BAT) activation [9–11] and beige adipocyte formation [3, 12] in cancer patients and tumor-bearing animal models increase energy consumption, which precedes adipose tissue and skeletal muscle atrophy [3].

According to NIH [13], in 2013, 86.8% of people aged 18 or older in the United States drank alcohol at some point in their life time. Alcoholics showed a significant increase in total cancer mortality in comparison with the general population [14]. Alcohol consumption is a known risking factor for cancers [15, 16]. As estimated, 5.2% of all male cancers and 1.7% of all female cancers are related to alcohol intake [17]. Alcohol is oxidized into acetaldehyde via alcohol dehydrogenase and alcohol-inducible cytochrome P-4502E1 (CYP2E1), and further oxidized to acetate by aldehyde dehydrogenase [18]. Because acetaldehyde is highly reactive, it is toxic and carcinogenic [19], which binds to DNA and forms carcinogenic adducts. Acetaldehyde-derived DNA adducts such as N^2^-ethylidene-2^’^-deoxyguanosine (N^2^-EtdG) block translesion DNA synthesis and induce mutations [20, 21]. Aldehydes also induces genomic instability [22]. Consistently, alcohol consumption increases the risk of colorectal cancer [23, 24].

Though the tumorigenic effects of alcohol have been well studied, its impacts on the metabolic state of cancer patients are far less defined. Chronic alcohol intake enhances the oxidative capacity of brown adipose tissue (BAT) [25–27]. In addition, we recently reported that alcohol consumption induces white adipose tissue browning and activates thermogenesis by elevating peripheral retinoic acid (RA) levels [28]. Due to the lower nutrient partitioning priority for adipose tissue and skeletal muscle, the increased energy demand from BAT and beige adipocytes induced by alcohol likely deprives nutrients available for muscle and adipose tissue of cancer patients, aggravating CAC. Using a mice model bearing melanoma tumors, the objective of this study was to analyze the impact of alcohol intake on browning of adipose tissues of tumor-bearing mice and its association with CAC.

## RESULTS

### Alcohol intake increases body weight and tissue mass loss in tumor-bearing mice

**Figure 1:**
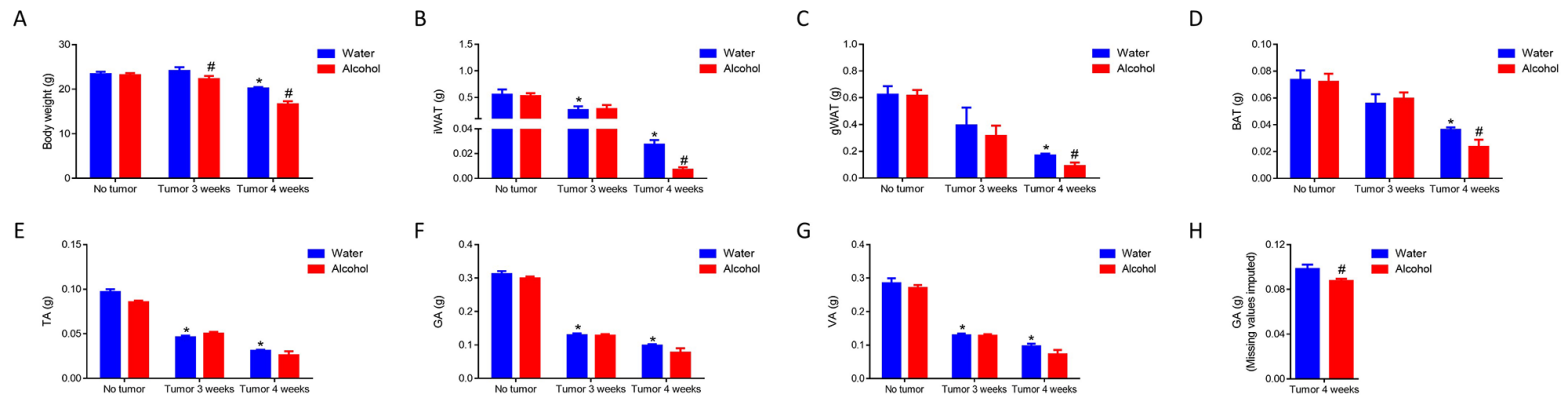
Alcohol increased body weight and tissue mass loss in mice implanted with tumor cells C57BL6 mice were supplemented with 0 or 20% (w/v) alcohol for 3 months, and then inoculated with B16BL6 melanoma cells for 3 or 4 weeks. **(A)** Body weight. **(B)** Inguinal white adipose tissue (iWAT) weight. **(C)** Gonadal white adipose tissue (gWAT) weight. **(D)** Brown adipose tissue (BAT) weight. **(E)** *Tibialis anterior* (TA) muscle weight. **(F)** *Gastrocnemius* (GA) muscle weight. **(G)** *Vastus lateralis* (VA) weight. **(H)** GA muscle weight after adding missing values of GA imputed according to its correlation with body weight. (^*^compare to the control mice, ^#^ compare to the mice fed with water, *p* < 0.05, mean ± SEM)

To explore the effects of chronic alcohol on CAC, C57BL/6 mice were fed with 0% or 20% (w/v) alcohol for 3 months, then inoculated with B16BL6 melanoma cells and continued to feed with/without alcohol for 3 or 4 weeks. Regardless of alcohol treatments, tumor cell inoculation reduced body weight and induced the loss of fat and muscle mass. Compared to tumor-bearing mice without alcohol, the alcohol group had lower body weight (Figure 1A), in line with the reduced mass of inguinal white adipose tissue (iWAT), gonadal white adipose tissue (gWAT), and BAT (Figure 1B–1D). Due to the large variations in weight loss among mice inoculated with tumors, the weights of *tibialis anterior* muscle (TA), *gastrocnemius* muscle (GA) and *vastus lateralis* muscle (VA) between mice with/without alcohol did not reach significance (Figure 1E–1G). In addition, because 4 mice in the alcohol plus tumor group were euthanized/died before the end of 4 weeks due to emaciation, their muscle tissues were not isolated and included in above calculation. GA muscle weight was linearly correlated to body weight (*p* = 0.01, R^2^ = 0.8); when the missing values were imputed using the regression equation, there was a significant reduction in GA muscle mass of alcohol treated tumor bearing mice (Figure 1H).

### Alcohol intake enhances white adipose tissue browning in tumor-bearing mice

**Figure 2:**
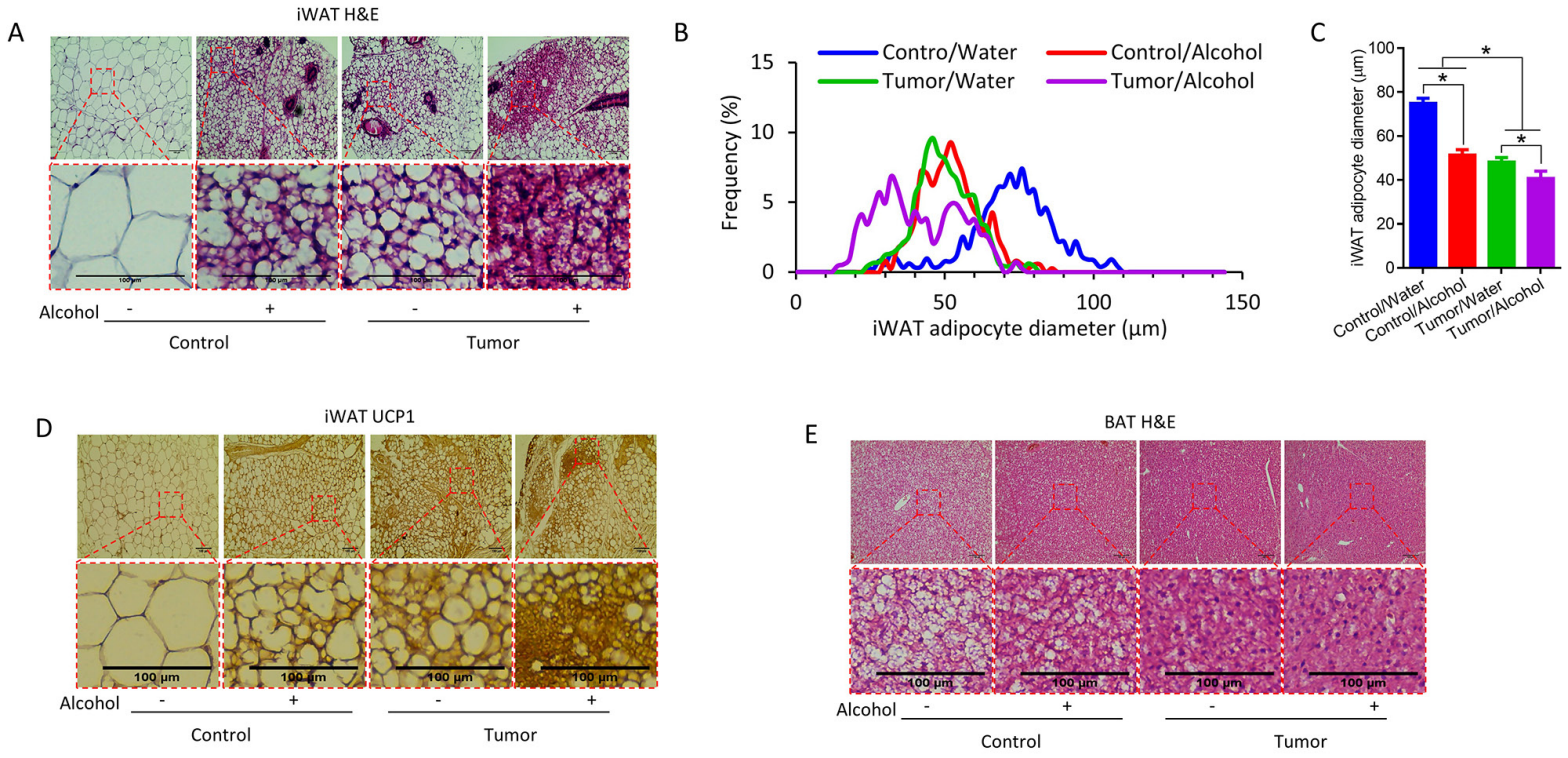
Alcohol enhanced white adipose tissue browning in tumor mice **(A)** Representative images of H&E stained inguinal white adipose tissue (iWAT). **(B)** iWAT adipocytes size distribution. **(C)** Average iWAT adipocyte diameter. **(D)** Representative images of iWAT adipocytes stained with UCP1. **(E)** Representative images of H&E stained BAT. Scale bar = 100 μm.

Consistent with a previous study [3], iWAT adipocytes became smaller and multilocular after tumor implantation (Figure 2A–2B). More UCP1 positive beige adipocytes were observed in tumor implanted mice. In addition, alcohol consumption further increased the abundance of multilocular adipocytes in the tumor-bearing mice with smaller adipocyte sizes (Figure 2A–2C). The UCP1 content in iWAT was increased due to both tumor implantation and alcohol consumption (Figure 2D). Similarly, the lipid content in BAT of tumor-bearing mice was lower than control mice, which was further reduced due to alcohol intake (Figure 2E).

**Figure 3:**
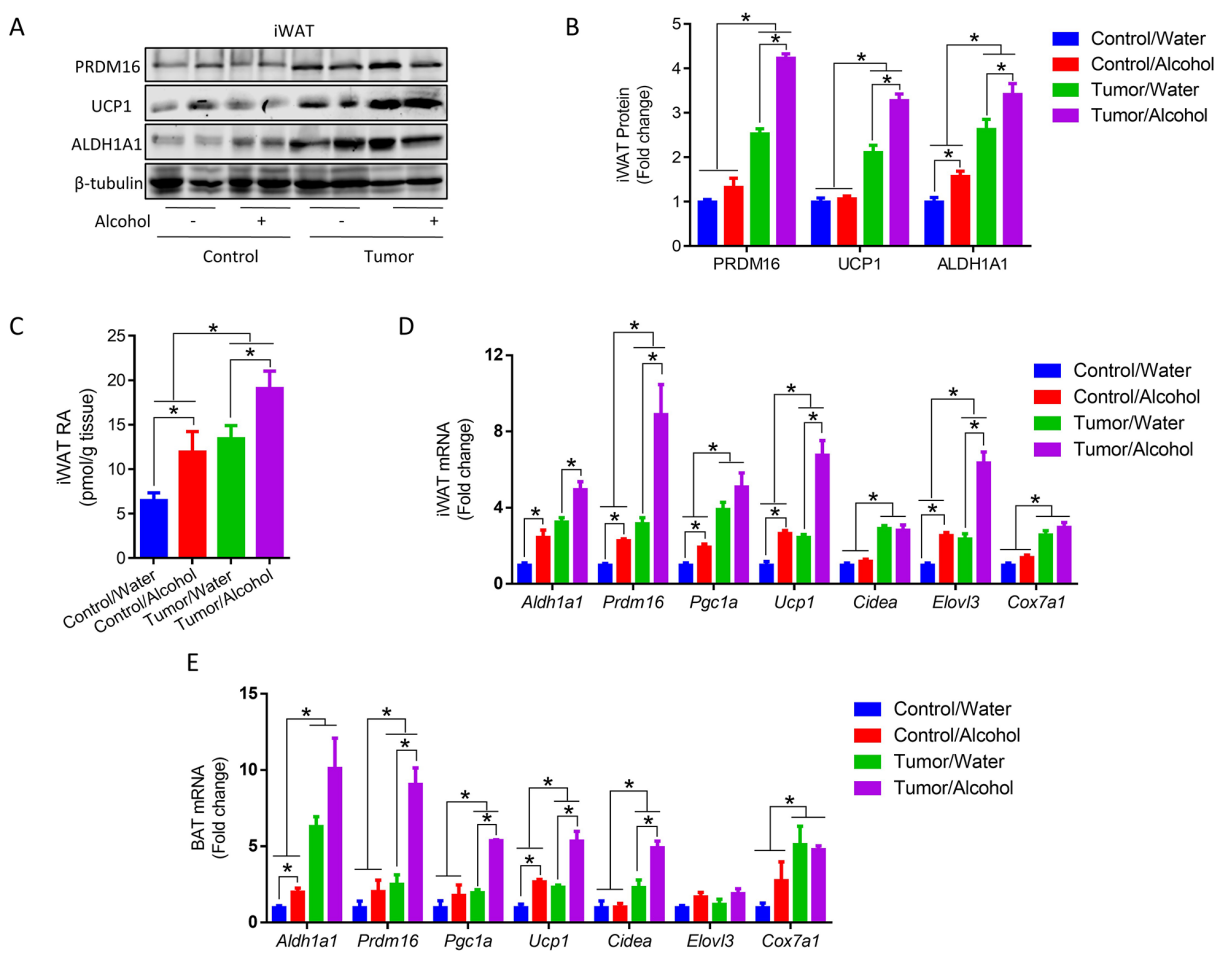
Alcohol and tumor inoculation upregulated brown/beige adipose genes **(A)** Protein contents of PRDM16, ALDH1A1 and UCP1 in iWAT. **(B)** Quantification of protein contents. **(C)** RA contents in iWAT. **(D)** mRNA levels of *Aldh1a1*, *Prdm16*, *Pgc1a*, *Ucp1*, *Cidea, Elovl3* and *Cox7a1* in iWAT. **(E)** mRNA levels of *Aldh1a1*, *Prdm16*, *Pgc1a*, *Ucp1*, *Cidea, Elovl3* and *Cox7a1* in BAT. (^*^p < 0.05, mean ± SEM).

ALDH1A1 plays an important role in producing RA and inducing beige adipogenesis [28]. Mice implanted with tumor cells had higher ALDH1A1 protein contents (Figure 3A and 3B). Alcohol consumption increased ALDH1A1 protein levels in both control and tumor mice compared to their water-drinking counterparts (Figure 3A and 3B). Furthermore, we analyzed RA in iWAT and found that tumor mice had higher RA level in iWAT and alcohol consumption increased RA in both control and tumor mice (Figure 3C). Consequently, alcohol increased PR/SET Domain 16 (PRDM16) and UCP1 protein contents in iWAT of tumor mice (Figure 3A and 3B). Similarly, alcohol increased the expression of *Aldh1a1* and the browning related genes including *Prdm16*, peroxisome proliferator-activated receptor gamma coactivator 1 alpha (*Ppargc1a*), *Ucp1*, cell death-inducing DNA fragmentation factor, alpha subunit-like effector A (*Cidea*), elongation of very long chain fatty acids (FEN1/Elo2, SUR4/Elo3, yeast)-like 3 (*Elovl3*) and cytochrome c oxidase subunit VIIa 1 (*Cox7a1*) (Figure 3D). In addition, tumor mice had higher mRNA levels of brown adipose genes in BAT, which was further upregulated by alcohol (Figure 3E).

**Figure 4:**
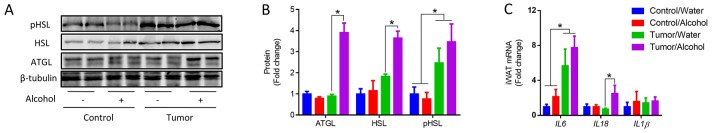
Alcohol increased lipolysis in inguinal white adipose tissue (iWAT) of tumor mice **(A)** Protein contents of pHSL, HSL, and ATGL in iWAT. **(B)** Quantification of protein contents. **(C)** mRNA levels of *IL6*, *IL18* and *IL1β* in iWAT. (^*^p < 0.05, mean ± SEM).

White adipose tissue browning leads to increased lipolysis. The protein levels of hormone sensitive lipase (HSL), phosphorylated HSL (Ser563) and adipose triglyceride lipase (ATGL) were higher in alcohol supplemented mice implanted with tumor (Figure 4A and 4B). In addition, chronic inflammation is known to increase UCP1 expression and browning of WAT in cancer mice [3] and lipolysis [29, 30]. We found that tumor implantation increased interleukin (IL) 6 expression in iWAT, while alcohol consumption increased IL18 expression in tumor-bearing mice (Figure 4C).

In summary, these data showed that tumor induced activation of BAT and browning of white adipose tissue, which increased lipolysis.

### Alcohol intake induces skeletal muscle protein degradation, apoptosis and inflammation

**Figure 5:**
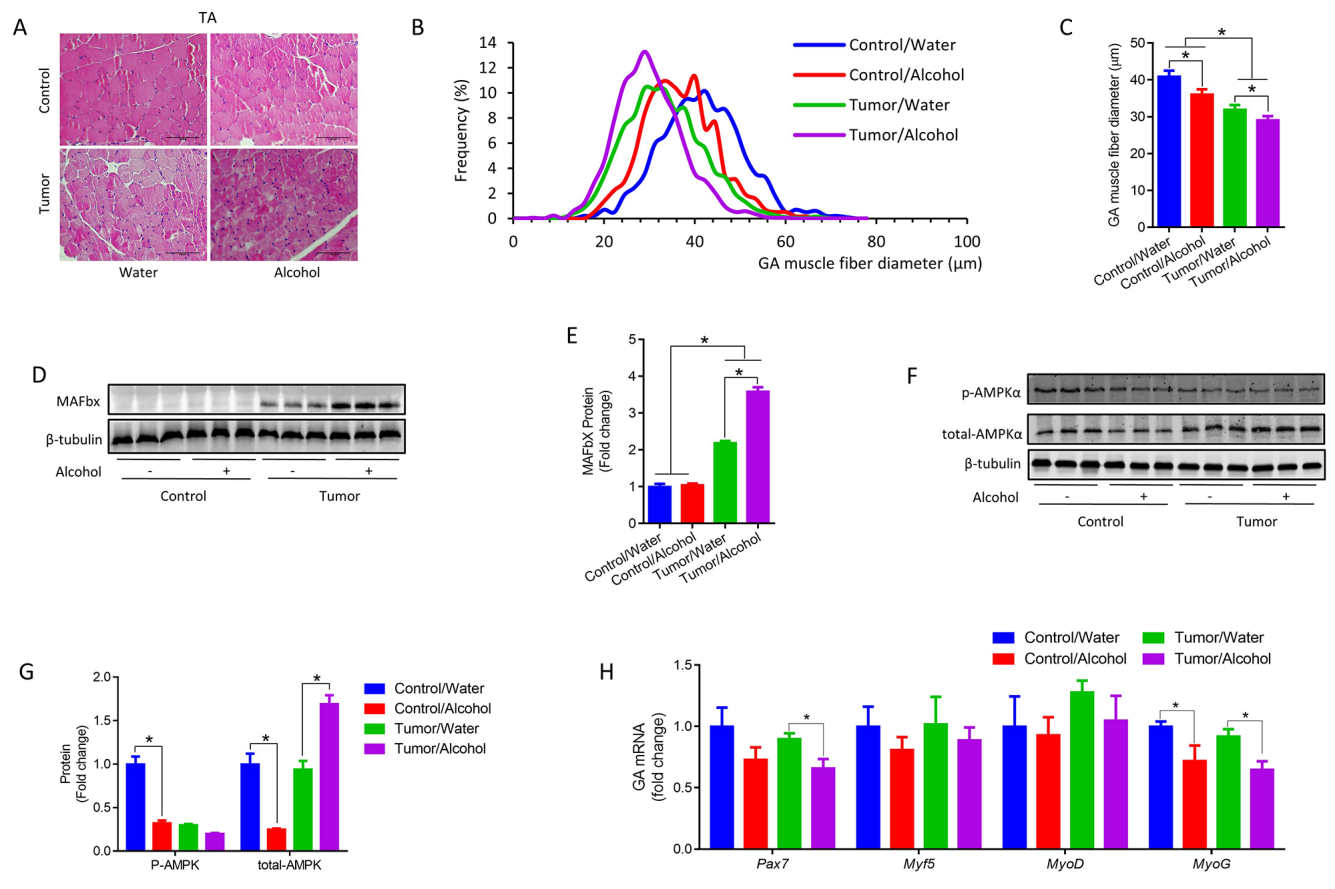
Alcohol promoted skeletal muscle atrophy in tumor mice **(A)** Representative images of H&E stained Tibialis anterior (TA) muscle, error bar = 100 μm. **(B)** Size distribution of TA muscle fibers. **(C)** Average diameter of TA muscle fibers. **(D)** Protein content of MAFbx in GA muscle. **(E)** Quantification of MAFbx protein content. **(F)** Protein content of pAMPK and total-AMPK in GA muscle. **(G)** Quantification of pAMPK and total-AMPK protein content. **(H)** mRNA levels of *Pax7*, *Myf5*, *MyoD* and *MyoG* in GA muscle. (^*^p < 0.05, mean ± SEM).

WAT browning is an early event in the pathophysiology of CAC, preceding skeletal muscle loss [3]. Tumor implanted mice had smaller fibers in TA showing the occurrence of muscle atrophy, and alcohol consumption aggravated muscle atrophy in tumor mice (Figure 5A–5C). Cachexia is associated with degradation of actomyosin, actin and myosin [31]. Here we found tumor cell implantation increased the protein content of muscle atrophy F-box (MAFbx) (Figure 5D and 5E), a muscle specific E3-ubiquitin ligase, indicating increased protein degradation in skeletal muscle. Alcohol further increased MAFbx expression (Figure 5D and 5E), which would aggravate muscle loss in mice with tumors. On the contrary, alcohol consumption impaired AMP-activated protein kinase (AMPK) activation (Figure 5F and 5G), which was important for myogenesis and muscle regeneration [32–34]. Consistently, alcohol reduced the expression of paired box 7 (*Pax7*) in GA muscle of tumor mice, indicating decreased satellite cell density; mRNA expression of myogenin (MyoG) was down-regulated in both control and tumor mice, suggesting impaired myogenesis and muscle protein synthesis (Figure 5H). These data are consistent with earlier reports showing that alcohol consumption inhibits muscle protein synthesis [35–37].

**Figure 6:**
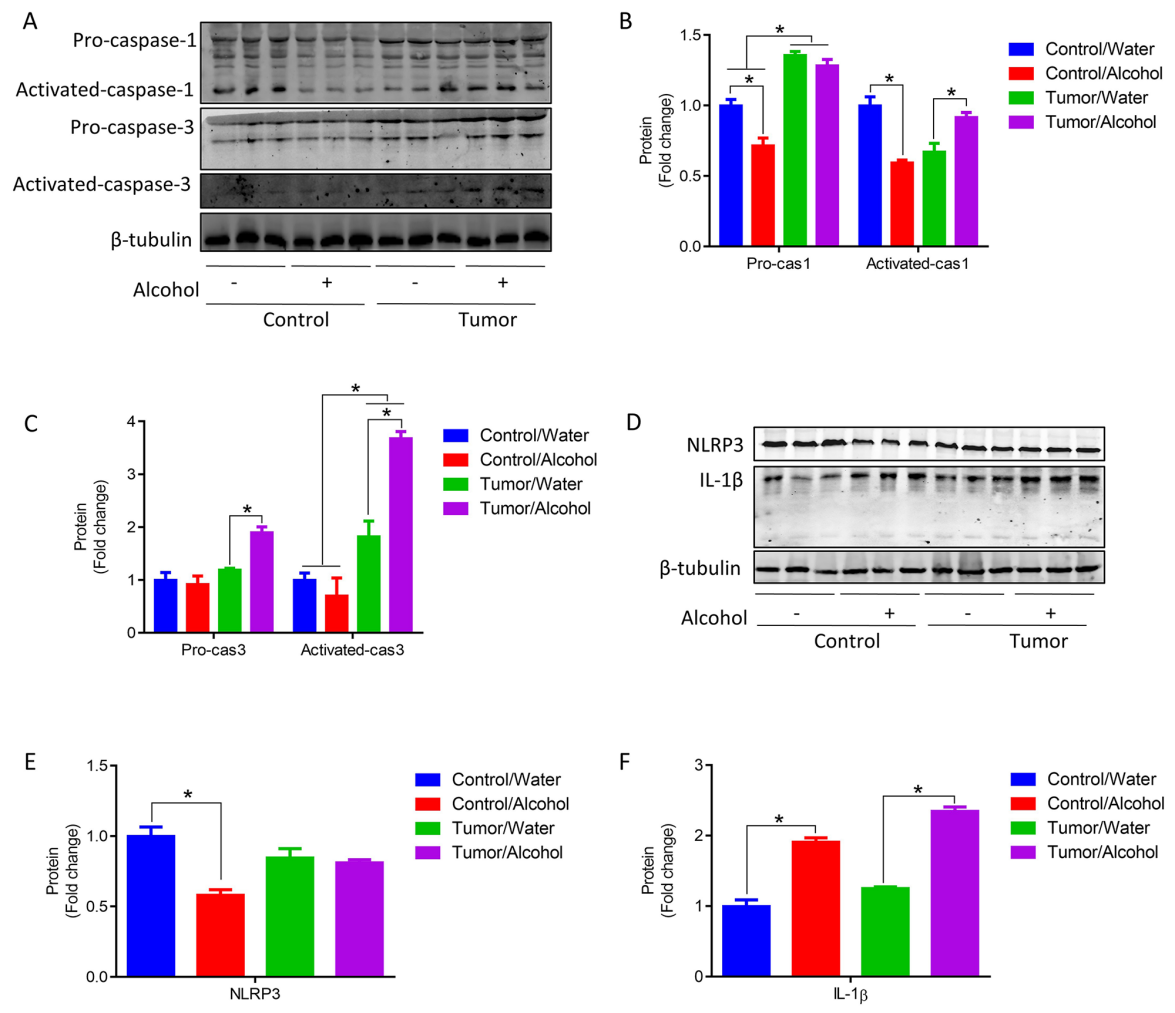
Alcohol promoted apoptosis and inflammation in skeletal muscle of tumor mice **(A-C)** Pro-caspase 1, activated caspase 1, Pro-caspase 3 and activated caspase 3 protein contents in GA muscle. **(D-F)** NLRP3 and IL-1β protein contents in GA muscle. (^*^p < 0.05, mean ± SEM).

In addition, alcohol reduced pro-caspase 1 but promoted caspase 1 activation in mice with tumors (Figure 6A and 6B). Activation of caspase-3 accelerates muscle proteolysis and loss [5, 38, 39]. Here we found that alcohol increased pro-caspase 3 and activated caspase 3 in mice with tumors (Figure 6A and 6C). Alcohol reduced PYD-domains-containing protein 3 (NLRP3) in control mice but not in mice with tumors (Figure 6D and 6E). Moreover, alcohol increased IL-1β in control and tumor mice (Figure 6D and 6F). In summary, these data suggest that chronic alcohol consumption increases protein degradation, cell apoptosis and inflammation in the skeletal muscle of mice with tumors.

## DISCUSSION

Alcohol and its metabolite, acetaldehyde, has long been recognized as carcinogens [15, 16, 19, 22]. However, the role of alcohol on energy homeostasis of cancer patients is poorly understood. In this study, we discovered that alcohol consumption aggravates cancer induced cachexia partially via inducing white adipose tissue browning. Our study revealed the relationship between alcohol and CAC which is significant for cancer treatment and patient care.

Alcohol and retinol (Vitamin A) share common metabolic enzymes involved in their conversion into aldehydes and acids [40]. We previously reported that alcohol consumption promotes adipose tissue browning by increasing the peripheral RA level in serum and adipose tissue [28]. In this study, we did not detect significant reduction in body weight and fat mass due to alcohol consumption in the absence of tumor, but reduction in body weight was observed in male mice due to alcohol consumption in our previous study [28]; the difference could be due to gender difference of mice and also the dose of alcohol used. In addition, we found that alcohol consumption upregulated ALDH1A1 which in turn elevated RA in adipose tissue. RA blocks white adipogenesis via inhibiting *Zfp423* expression [41]; on the other hand, RA upregulates the expression of brown adipogenic genes, activates BAT and induces the formation of thermogenic beige adipocytes [28, 42–44]. While the thermogenic activity of brown and beige adipocytes increases energy expenditure which contributes to obesity prevention, it causes unfavorable consequences in cancer patients [2, 3, 12]; the elevated energy expenditure and body weight loss increase mortality [45]. Currently, RA is commonly used in cancer prevention and treatment [46–48], and our data suggest the negative impact of RA treatment in dissipating energy needed for patients, especially those with late stage cancers.

WAT browning is an early event in the pathophysiology of CAC, preceding skeletal muscle loss [3]. In this study, mice implanted with tumor lose more than 90% WAT and more than 60% skeletal muscle in 4 weeks, with alcohol consumption further worsening muscle and fat loss. Alcohol is well known to inhibit muscle protein synthesis [35–37]. In the current study, at the molecular level, alcohol promoted muscle protein degradation, apoptosis and inflammation. Cancer cachexia is characterized by systemic inflammation. The elevated cytokines induce anorexia, upregulate expression of muscle atrophy-associated genes and increase protein degradation [2, 5, 49–51]. Catabolism of muscle protein increases while anabolism of proteins decreases which result in net protein breakdown [5]. Consistently, alcohol consumption upregulated IL-1β, a marker of inflammation; alcohol also activated Caspase-1 and Caspase-3, key mediators of cell apoptosis. In addition, alcohol consumption dramatically increased MAFbx, a muscle-specific E3 ubiquitin ligases that mediates muscle protein degradation [31, 52]. In addition, alcohol consumption reduced the satellite cell density and inhibited myogenesis, which impair muscle regeneration and lead to muscle loss.

Though CAC is known to reduce life quality and survival time, its treatment options are limited [53]. In addition to provide nutrients, therapies reducing energy expenditure may also be needed for effective CAC management. In agreement, in a mouse model, inhibition of catabolic lipase by genetic ablation of *Atgl* or *Hsl* ameliorates CAC [54]. Based on our discovery, to improve life quality and prolong survival time of cancer patients, alcohol consumption needs to be controlled in order to reduce energy dissipation.

## MATERIALS AND METHODS

### Animal treatments and tumor cell inoculation

Female C57BL/6 mice at 6 weeks of age were purchased from Charles River Laboratories (Wilmington, MA) and single housed in plastic cages with micro-filter tops in the SPBS Vivarium at WSU-Spokane, which is fully accredited by the Association for the Assessment and Accreditation of Laboratory Animal Care. Mice were allowed free access to Purina 5001 rodent laboratory chow and sterilized Milli-Q water. After one week of acclimation, mice were randomly divided into two groups. The control group was continuously provided with chow and Milli-Q water. The treatment group was provided with chow and 20% (w/v) alcohol diluted from 190-proof Everclear (St. Louis, MO) with sterilized Milli-Q water. Similar doses of alcohol have been used in our previous studies [28, 45, 55]. After 3 months, mice in each group were further divided into two sub-groups with/without tumor inoculation. All studies were approved by the Institutional Animal Care and Use Committee at Washington State University.

For tumor inoculation, highly invasive and metastatic B16BL6 melanoma cell line originally obtained from the Mason Research Institute, Worcester, MA, was used. Tumor cells were cultured in Dulbecco’s modified Eagles medium supplemented with 10% FBS, 1% penicillin and streptomycin in a humidified incubator with 5% CO_2_ at 37°C. Cells were harvested when they reached 50-70% confluence. Each mouse was inoculated with 2×10^5^ cells (in 200 μl PBS) subcutaneously in the right side of the hip.

Animals were euthanized at 3 or 4 weeks after tumor inoculation. There were originally 10 mice for control and 4-weeks tumor groups. However, for the tumor inoculated mice consuming alcohol, four mice were euthanized/died during the 4^th^ week due to emaciation. These mice were stored in -20 °C before fat tissues were isolated and muscle tissues were not isolated. To balance, six samples from other three groups were randomly chosen for further biochemical analysis. Muscle tissues used for biochemical analysis were isolated at 4 weeks after tumor inoculation. Because of limited amount of fat tissues in mice inoculated with tumor for 4 weeks, fat tissues used for biochemical analysis were isolated at 3 weeks after tumor inoculation.

### Tissue processing and histological examination

Adipose and muscle tissues were processed as previously described [42]. Briefly, tissues were fixed in 4% paraformaldehyde for 12 h at 4 °C, then paraffin embedded and sectioned. Following deparaffinization, tissue sections were used for H&E staining or immunostaining. For immunostaining, sections were heated in citrate buffer for 20 min, blocked with 5% goat serum in TBS containing 0.3% Triton X-100 for 2 h, then incubated sequentially with primary antibodies overnight and secondary antibodies for 1 h. Sections were then mounted in a BAPI mounting medium (Vector Lab, Burlingame, CA) and subjected to microscopic examination.

### Quantitative real-time PCR (qRT-PCR)

Total RNA was isolated using TRIzol (Life technologies, Grand Island, NY), and cDNA was synthesized using iScript™ cDNA Synthesis Kit (Bio-Rad, Hercules, CA). qRT-PCR was performed using CFX RT-PCR detection system (Bio-Rad) with SYBR green RT-PCR kit from Bio-Rad (Hercules, CA). Primer sequences are listed in Table 1. All values were normalized to the level of a housekeeping gene: 18S rRNA.

**Table 1:** qPCR primer sequences

Gene name	Forward (5’ to 3’)	Reverse (5’ to 3’)	Product size (bp)	Accession number
*Prdm16*	CAGCACGGTGAAGCCATTC	GCGTGCATCCGCTTGTG	87	XM_006539178.3
*Ucp1*	ACTGCCACACCTCCAGTCATT	CTTTGCCTCACTCAGGATTGG	123	NM_009463.3
*Pgc1a*	CCTCACACCAAACCCACAGA	CCTCATGCGGTCACTGTCG	272	XM_006503779.3
*Cidea*	ATCACAACTGGCCTGGTTACG	TACTACCCGGTGTCCATTTCT	136	NM_007702.2
*Elovl3*	GATGGTTCTGGGCACCATCTT	CGTTGTTGTGTGGCATCCTT	73	XM_006526624.3
*Cox7a1*	CAGCGTCATGGTCAGTCTGT	AGAAAACCGTGTGGCAGAGA	112	NM_009944.3
*Aldh1a1*	CCTTGCATTGTGTTTGCAGATG	GCTCGCTCAACACTCCTTTTC	158	NM_013467.3
*IL-1β*	TCGCTCAGGGTCACAAGAAA	CATCAGAGGCAAGGAGGAAAAC	73	XM_006498795.3
*IL-18*	ATGCTTTCTGGACTCCTGCCTGCT	GGCGGCTTTCTTTGTCCTGATGCT	89	XM_006510028.3
*IL-6*	GAGGATACCACTCCCAACAGACC	AAGTGCATCATCGTTGTTCATACA	141	NM_001314054.1
*Pax7*	TTGGGGAACACTCCGCTGTGC	CAGGGCTTGGGAAGGGTTGGC	115	XM_006538631.1
*Myf5*	AAACTCCGGGAGCTCCGCCT	GGCAGCCGTCCGTCATGTCC	125	XM_006513319.2
*MyoD*	TCTGGAGCCCTCCTGGCACC	CGGGAAGGGGGAGAGTGGGG	100	NM_010866.2
*MyoG*	GAGATCCTGCGCAGCGCCAT	CCCCGCCTCTGTAGCGGAGA	97	NM_031189.2
18s rRNA	TTGTACACACCGCCCGTCGC	CTTCTCAGCGCTCCGCCAGG	102	NR_003278.3

### Immunoblotting analyses

Immunoblotting analysis was performed as previously described [42] using an Odyssey Infrared Imaging System (LiCor Biosciences, Lincoln, NE). Density of bands was quantified and then normalized to the β-tubulin content.

### Antibodies and reagents

Antibodies against β-tubulin (#2146), IL-1β (#12242), caspase-1 (#3866), cleaved caspase-1 (#4199), caspase-3 (#9662), cleaved caspase-3 (#9661), HSL (#4107), phospho-HSL (#4139), ATGL (#2138), AMPKα (#2532) and phosphor-AMPKα (#2535) were purchased from Cell Signaling (Danvers, MA). Antibodies against UCP1 (Cat. No. PA1-24894) and PR/SET Domain 16 (PRDM16) (#PA5-20872) were bought from TheromoFisher Scientific (Waltham, MA). Antibody against PYD-domains-containing protein 3 (NLRP3) (#PA1665) was purchased from Boster Biological Technology (Fremont, CA). Antibody against muscle atrophy F-box (MAFbx) (#sc-33782) was purchased from Santa Cruz Biotechnology (Dallas, Texas). IRDye 800CW goat anti-rabbit and IRDye 680 goat anti-mouse secondary antibodies were bought from LI-COR Biosciences (Lincoln, NE).

### High performance liquid chromatography (HPLC) assay

Toprevent isomerization and degradation of retinoids, serum samples were stored at -80 °C and protected from light. RA was extracted according to a published protocol [56] and measured by HPLC [28] using a reverse phase column (Luna 3 μm C18(2) 100 Å, LC Column 150 x 3 mm). The mobile phase, methanol/H2O (65/35) was pumped at 1.0 mL/min. RA was detected by a Nexera X2 diode array detector at a wavelength of 340 nm. RA standard was used for calculating standard curves and recovery rates, and the concentration of RA was expressed as pmol/mg wet tissue weight.

### Statistical analysis

All data are presented as means ± standard errors of the mean (SEM). One-way ANOVA followed by a post hoc Tukey’s test was used to compare differences among multiple groups. An unpaired, two-tail Student’s t-test was used to compare differences between two groups. All analyses were performed using SAS 9.0 (SAS Institute Inc., Cary, NC). Statistical significance was assigned at *p* < 0.05.
